# Healthcare-associated infections in home healthcare: an extensive assessment, 2019

**DOI:** 10.2807/1560-7917.ES.2021.26.5.1900646

**Published:** 2021-02-04

**Authors:** Ana Hoxha, Els Duysburgh, Laure Mortgat

**Affiliations:** 1 European Programme for Intervention Epidemiology Training (EPIET), European Centre for Disease Prevention and Control (ECDC), Stockholm, Sweden; 2 Department of Epidemiology and Public Health, Sciensano, Brussels, Belgium

**Keywords:** Healthcare-associated infections, home care, home healthcare, surveillance, infection prevention and control, HAI, ICP, recommendations

## Abstract

**Introduction:**

The number of patients and clinical conditions treated in home healthcare (HHC) is increasing. Care in home settings presents many challenges, including healthcare-associated infections (HAI). Currently, in Belgium, data and guidelines on the topic are lacking.

**Aim:**

To develop a definition of HAI in HHC and investigate associated risk factors and recommendations for infection prevention and control (IPC).

**Methods:**

The study included three components: a scoping literature review, in-depth interviews with individuals involved in HHC and a two-round Delphi survey to reach consensus among key informants on the previous steps’ results.

**Results:**

The literature review included 47 publications. We conducted 21 in-depth interviews. The Delphi survey’s two rounds had 21 and 23 participants, respectively. No standard definition was broadly accepted or known. Evidence on associated risk factors was impacted by methodological limitations and recommendations were inconsistent. Agreement was reached on defining HAI in HHC as any infection specifically linked with providing care that develops in a patient receiving HHC from a professional healthcare worker and occurs ≥ 48 hours after starting HHC. Risk factors were hand hygiene, untrained patients and caregivers, patients’ hygiene and presence and management of invasive devices. Agreed recommendations were to adapt and standardise existing IPC guidelines to HHC and to perform a national point prevalence study to measure the burden of HAI in HHC.

**Conclusions:**

This study offers an overview of available evidence and field knowledge of HAI in HHC. It provides a framework for a prevalence study, future monitoring policies and guidelines on IPC in Belgium.

## Introduction

In an ageing society, where the prevalence of chronic diseases is increasing and leading to new, advanced and often complex medical treatments, demand for healthcare is constantly rising. Considered the most frequent adverse event (AE) in healthcare delivery worldwide [1], healthcare-associated infections (HAI) are a public health threat. The burden of HAI is considerable, including not only mortality and morbidity, but also financial and socio-economic costs due to increased length of hospitalisation; additional need for diagnostics, treatments and rehabilitation of infected patients; and loss of productivity and quality of life [2-4]. HAI have therefore become a priority in many countries’ political health agendas [5]. However, as highlighted by the World Health Organization (WHO), while the focus is often on inpatient settings, no healthcare setting is exempt from the risk of HAI.

In Belgium, as in other European and western countries, a considerable shift from inpatient care, provided in hospitals, to outpatient care, including home healthcare (HHC), has been observed in recent years [6,7]. HHC is defined as care provided by professionals to a person at their own home and covers a wide range of activities, from regular routine check-up visits to end-of-life care [8]. In Belgium, HHC is prescribed by a medical doctor in a hospital, a private practice or other health facility and is provided to the patient by a general practitioner, private nurses or other healthcare workers (e.g. physiotherapist) who are either self-employed or employed by an organisation dedicated to providing HHC. Regardless of the provider, if prescribed, HHC is reimbursed by the Belgian health insurance system. The number of patients receiving HHC services has been increasing and is expected to continue to increase. While healthy, elderly patients have always received regular routine care at home, ill patients receiving more complex care are now increasingly transferred to their homes, with the hope of gaining quality of life and reducing healthcare costs [9,10]. However, receiving care at home poses new challenges and exposes patients to other risks, including HAI.

In recent decades, awareness of HAI has led to extensive research worldwide, which has identified risk factors and proposed measures and interventions to prevent or mitigate the consequences of HAI in inpatient care [11-14]; however, studies on HAI in HHC remain scarce. At present, most of the knowledge used in infection prevention and control (IPC) practice in HHC originates from the evidence collected in hospitals. In Belgium, there are currently several national surveillance systems for HAI in hospital setting [15]. In 2017, it was estimated that 7% of patients admitted to hospital contracted a HAI [16]. However, data on HAI for HHC are missing, as are specific IPC guidelines and measures.

This study aimed to conduct a conceptual analysis of HAI in HHC, with the following objectives: (i) develop a definition for HAI in HHC, (ii) identify specific risk factors for HAI acquisition in HHC settings and (iii) develop a standardised framework for prevention and control of HAI in HHC in Belgium.

## Methods

### Study design

To address the study objectives, we performed a scoping literature review, in-depth interviews and a two-round Delphi survey.

The scoping literature review included peer-reviewed papers and grey literature written in English, Dutch, French, German, Italian and Spanish, conducted on patients receiving HHC, published in high-income countries after 1999 and relevant to at least one of the three aspects defined in the study objectives. We used PubMed, Embase, Science Direct, the Cumulative Index to Nursing and Allied Health Literature and Cochrane Library to search for peer-reviewed papers, and Google Scholar, Open Grey, the Networked Digital Library of theses and Dissertations, and Grey Literature Report to find grey literature. We screened the websites of WHO, the United States (US) Centres for Disease Control and Prevention, the European Centre for Disease Prevention and Control (ECDC), the Belgian Healthcare Knowledge Centre and the Belgian Superior Health Council and conducted hand searching of references to identify relevant cross citations. The search strategy and exact strings used are presented in Supplement I.

We conducted in-depth interviews with key informants in Belgium, including healthcare professionals who were carrying out home visits or were involved in healthcare policy, HHC management or projects on hospitalisation at home. Key informants were selected using the purposive sampling method [17]. To guide the identification and selection of eligible key informants, we developed an overview of different profiles we wanted to target (Supplement II). We qualified these profiles based on their function or job title and their work experience. It was important to include individuals who were familiar with organisational, management and/or policy concepts regarding HHC and HAI so that they would be able to address the study objectives. Key informants were selected from all three Belgian regions: Brussels-capital, Flanders and Wallonia. We aimed to conduct a total of 30 interviews; however, this number could be lowered during the study if the information being collected was no longer adding new insights. Two of the study researchers (ED and LM) conducted recruitment and interviewed key informants, starting with individuals they already knew through their networks. Additional participants were recommended by this first group (snowball effect) or by colleagues, and others were identified though web-based searches performed using keywords such as ‘HHC in Belgium’, ‘hospitalisation at home projects’ and names of Belgian organisations providing HHC. An interview guide was developed and used during the interviews (Supplement III).

The Delphi survey consisted of two rounds. The first questionnaire was developed based on the results of the scoping review and the in-depth interviews, while the second was based on the results of the first round (Supplement IV). The Delphi survey was conducted online, using LimeSurvey, and targeted the same key informants as the in-depth interviews.

### Data collection and analysis

For the scoping literature review, predetermined relevant characteristics of the selected articles were extracted and a self-developed quality assessment tool was used to evaluate them (Table 1).

**Table 1:** Evaluation criteria for quality assessment and scoring for each article included in the study

Characteristics	Evaluation criteria and scoring
Study information	**Study type** + + + experimental study/literature review + + observational study + expert opinion/essay
	**Setting** + + + study conducted in Belgium + + study conducted in Europe + study was not conducted in a high-income country^a^
	**Population** + + + all age groups + + only children/elderly + undefined
Source of infection	**Possible infection sites, as defined by ECDC [** 47 **]** + + + HAI in HHC + + presumed HAI in HHC + mixed source − undefined source
Contents and findings	**Definition of HAI in HHC** + + + provides definition + + uses accepted recognised definition (guidelines) + uses accepted definition (literature: peers) − no definition provided
	**Risk factors of HAI in HHC** + + + identifies risk factors + + lists risk factors + mentions risk factors − no risk factors provided
	**Recommendations for IPC** + + + identifies recommendations + + lists recommendations + mentions recommendations − no recommendations provided

HAI: healthcare associated infection; HHC: home healthcare; IPC: infection prevention and control.

^a^ Using World Bank criteria: https://data.worldbank.org/country/XD.

We attributed a score for each characteristic based on relevance to the study objectives ( +  +  +: matches completely/is completely fulfilled;  +  +: matches partially/partly fulfilled;  +: matches incompletely but sufficiently/is only partly but sufficiently fulfilled; −: does not match or matches insufficiently/is insufficiently fulfilled; c.b.e.: cannot be evaluated).

The characteristics to be evaluated were selected based on information the researchers considered relevant to the study objectives, and a scoring system was agreed upon. The articles with characteristics that scored higher were considered more appropriate for the purposes of the study and their results contributed more to the summary of the findings.

In-depth interviews were conducted by phone (by ED and LM), in the participants’ native languages, in January and February 2019. They were audio recorded and interview notes were taken. For the data analysis we used the deductive framework approach, in which the research objectives are used to group the data and then look for similarities and differences [18,19]. Immediately after the interviews were conducted, the data were grouped by the following themes: (i) the interviewees’ knowledge of definitions and prevalence, (ii) notification to healthcare authorities, (iii) risk factors, (iv) IPC management and measures and (v) availability of IPC guidelines, all of which were in the context of HAI in HHC.

The Delphi survey was conducted in March and April 2019. Level of agreement was measured and calculated using a four-point Likert scale ranging from 1 (“*Strongly disagree/very low importance”)* to 4 (“*Strongly agree/very high importance”*), as well as the option “*I don’t know”.* Consensus was defined as a minimum of 80% of the respondents agreeing or strongly agreeing (Likert scale 3 and 4) with the statement. The “*I don’t know*” answers were excluded from the calculation and did not contribute to the denominator of the consensus percentage. Among the statements that achieved consensus, the mean was calculated and used to determine the level of importance. Statements for which consensus was reached in the first round of the survey were excluded from the second round. If contradictory statements achieved consensus in the first round, or if no consensus was reached, the statement was assessed again in the second round. For risk factors only, even statements for which consensus was reached in the first round were further assessed in the second round in order to identify the five most necessary and five most feasible to act on.

The key informants selected to participate in the Delphi survey received the invitation to participate through an email that included a link to the questionnaire. Participation was voluntary and anonymous; therefore, we only collected information about area of work, years of expertise and knowledge of the topic from those who participated.

Data analysis was performed with STATA 14.

### Ethical statement

We did not need ethical approval for the implementation of this study. Written informed consent was obtained from all key informants before enrolment in the in-depth interviews. Participation in the interview was voluntary and confidentiality of the interviewees was protected and guaranteed. The Delphi survey was voluntary and anonymous.

## Results

### Scoping literature review

The search strategy, run on 30 October 2018, identified 3,171 peer-reviewed articles and six grey literature publications, from which 47 met the study inclusion criteria (Figure 1). Thirteen publications were articles written by experts that presented the state of the art or discussed best practices or guidelines, four were official guidelines and one was a draft of definitions for a surveillance system. The remaining 29 publications were research articles: 13 retrospective observational investigations, seven surveys, three literature reviews, three cohort studies, two point prevalence studies (PPS) and one randomised clinical trial.

**Figure:**
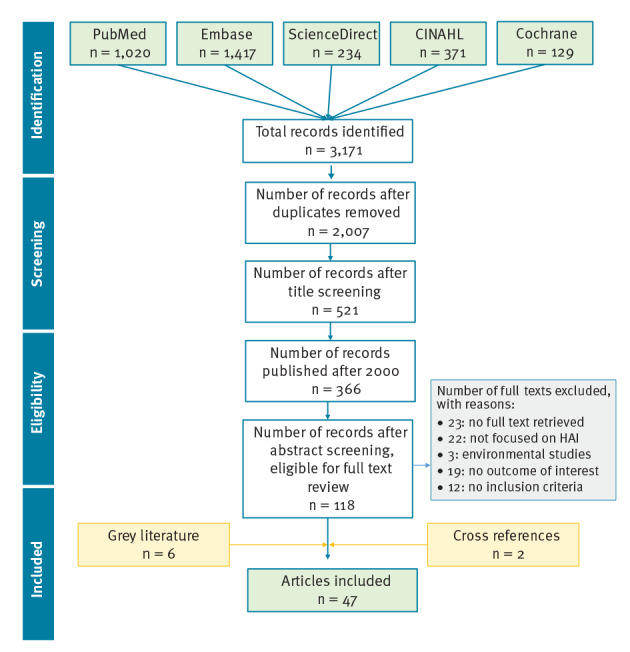
Flow diagram of the literature search and article selection process, 30 October 2018

Cochrane: Cochrane Library; CINAHL: Cumulative Index to Nursing and Allied Health Literature.

Among the 47 publications included in the study, 22 were from the US, 16 were from Europe, six were from Canada, one was from Hong Kong, one was from Saudi Arabia and one was not country specific and was published by WHO. Twenty-five publications focused on general unspecified HAI, or on multiple types of infection together, while 20 dealt specifically with catheter-related infections and two with ventilator-associated pneumonia. An overview of the results of the literature review is shown in Supplement V.

### Characteristics of in-depth interview and Delphi survey participants

A total of 20 healthcare professionals (nurses, general practitioners, a microbiologist and a physiotherapist) and one family caregiver participated in the in-depth interviews. Eleven interviews were conducted in Dutch and 10 in French, with each lasting 19–46 min. About half of the invited candidates agreed to be interviewed and we stopped enrolling participants when saturation of information was reached. A detailed description of the participants is provided in Supplement VI.

The response rate in the first and second round of the Delphi survey was 21/43 and 23/42, respectively. The two groups had an average of 14 years and 17 years of work experience in healthcare, respectively, of which more than half was specifically in HHC. The professional activities that were most represented were IPC, HHC practice and management of an organisation offering HHC services and policymaking.

### Definition of healthcare-associated infection in home healthcare

The definitions we encountered in the literature were heterogeneous. The Association for Professionals in Infection Control and Epidemiology (APIC) definition [20] was the one most used. The definition by Miliani et al., which combines elements of the APIC definition with ECDC case definitions for HAI in hospitals, was used in a study in France to measure the prevalence of HAI in HHC [21].

During the in-depth interviews, most of the interviewees said they had never encountered or used a specific definition of HAI in HHC. A few referred to the APIC definition.

Table 2 includes the definitions for which a consensus was reached in the first round of the Delphi survey and the final definition selected after the second round. After the two rounds, the agreed upon definition of HAI in HHC was detailed in its components and flexible to include different site infections.

**Table 2:** Two-round Delphi survey results on definition of healthcare-associated infections in home healthcare, by survey rounds, Belgium, April 2019

Definitions	Agreed and strongly agreed				
	Round 1 (N = 21)		Round 2 (N = 23)		
	%	n/N		%	n/N
Any infection that develops in a patient who receives HHC from a professional healthcare worker and that occurs 48 hours or later after initiating this HHC	86	18/21	35		8/23
Any infection that can be specifically linked with providing care (e.g. wound infection, infection linked with the use of catheters) that develops in a patient who receives HHC from a professional healthcare worker and that occurs 48 hours or later after initiating this HHC	90	18/20	65		15/23

HHC: home healthcare; N: total number of participants who replied; n: number of people who selected the options *“Agree”* or *“Strongly agree”.*

Respondents reached consensus for the definition highlighted in grey in the first round of the Delphi survey and this definition was also the most selected in the second round. Responses of *“I don’t know”* were not included in the calculations.

### Risk factors for healthcare-associated infection in home healthcare

Various risk factors emerged from the literature review; some were specific to a certain type of infection (e.g. central-line associated bloodstream infections, catheter-associated urinary tract infections), while others were more general. The methodology through which risk factors were identified varied, and included multivariable analyses on datasets of patients in HHC who developed HAI [22], literature review [23-25], environmental studies [26,27] and expert opinions [28-33]. As reported by Shang et al. in their systematic literature review of the risk factors of HAI in HHC, the identified studies are limited by small sample size and other methodological limitations, which in their case did not allow for a meta-analysis [23].

During the in-depth interviews, the vast majority of interviewees stated that risk factors for HAI at home were different than in hospital and classified them in three main categories: (i) patient’s lifestyle, living environment and socioeconomic status; (ii) patient’s characteristics and pathology; and (iii) the care provided.

Several risk factors were selected based on the literature review and in-depth interview findings and were presented in the Delphi survey. Respondents agreed that an action to control risk factors is needed and feasible for the following: hand hygiene, patients’ personal hygiene, training of patients and caregivers about measures to prevent HAI in HHC and presence and management of invasive devices (Table 3).

**Table 3:** Two-round Delphi survey results on risk factors for healthcare-associated infections in home healthcare, by survey round, Belgium, March and April 2019

List of risk factors	Round 1 (N = 21)		Round 2 (N = 23)	
	Agreed and strongly agreed		Most necessary to act on	Most feasible to act on
	%	n/N	n	n
Patient’s personal hygiene	100	20/20	15	15
Home hygiene	75	15/20	NA	NA
Home infrastructure (presence of sanitations, soap)	100	20/20	11	10
Presence of pet in the home environment	63	12/19	NA	NA
Education level of the patient	74	14/19	NA	NA
Presence of caregiver(s) in the household	63	12/19	NA	NA
Socio-economic status of the patient	84	16/19	3	1
Training of patient and caregiver(s) about the measures to prevent HAI in HHC	95	18/19	16	15
Patient’s age	81	17/21	0	1
Patient’s sex	14	3/21	NA	NA
Underlying health condition(s) of the patient	100	21/21	4	1
Medical condition for which HHC was indicated	95	19/20	1	1
Presence of invasive devices	100	21/21	14	6
Duration of the presence of invasive devices	100	21/21	11	10
Duration of HHC	81	17/21	0	2
Hand hygiene of Healthcare provider	95	19/20	16	18
Management of invasive devices by the healthcare provider	100	20/20	11	16
Frequency of visits by the healthcare provider	90	18/20	1	4
Lack of time by the healthcare provider during the visit	80	16/20	7	6
Communication between different care providers	74	14/19	NA	NA

HAI: healthcare-associated infection; HHC: home healthcare; N: total number of participants that replied; n: number of people who selected the options *”Agree”* or *“Strongly agree”*; NA: not applicable, as this statement/question was not asked in the second round because consensus was not reached (≥ 80% agreement) in the first round.

Respondents reached consensus in the first round for the risk factors highlighted in grey; four more selected in the second round are also highlighted. In the second round, participants could select five answers for the most necessary and the most feasible to act on. Responses of *“I don’t know”* were not included in the calculations.

### Measures and guidelines to prevent healthcare-associated infection in home healthcare

The vast majority of the studies included in the literature review (43/47) provided some kind of recommendations, focusing either on prevention and control of HAI in HHC or more broadly on the improvement of the standardisation, reporting and benchmarking of HAI in HHC across different countries. Several studies highlighted the need to raise awareness of HAI in HHC among patients and their caregivers, as well as to empower and train patients and caregivers on the measures and practices that can contribute to safe and HAI-free HHC [21,23,34-36].

Interviewees mentioned that the basic principles of IPC for HAI in hospitals and HHC were the same; nevertheless, their implementation in the home setting appears to be more challenging. The management of HAI in HHC is generally neglected compared with HAI prevention and control in hospitals and nursing homes, and the people working in the field of HHC feel the lack of general standardised guidelines.

In the first round of the Delphi survey, respondents reached consensus for all of the suggested measures to prevent and control HAI in HHC. In particular, all respondents agreed on the need for standardised IPC guidelines for HHC, available and accessible to all staff involved in HHC, with room for adaptation to the local context. As shown in Table 4, in the second round of the survey, a vast majority of respondents indicated that existing national and international accepted IPC guidelines for HAI and for specific technical procedures can be used in HHC, as long as adaptation to the home setting—when needed—was encouraged and possible.

**Table 4:** Second round of two-round Delphi survey results on measures to prevent and control healthcare-associated infection in home healthcare, Belgium, April 2019

Statements	Agreed and strongly agreed (N = 23)	
	%	n/N
Existing national and international accepted IPC guidelines for HAI (e.g. WHO guidelines on hand hygiene) can be used in HHC without adaptation	32	6/19
Existing national and international accepted IPC guidelines for HAI (e.g. WHO guidelines on hand hygiene) can be used in HHC, but need to be adapted to the home setting when needed	95	19/20
Existing national and international accepted IPC guidelines for HAI (e.g. WHO guidelines on hand hygiene) cannot be used in HHC, which requires specific guidelines	24	4/17
Existing national and international guidelines for specific technical procedures (e.g. hospital guidelines for preventing central line-associated bloodstream infection) can be used in HHC without adaptation	26	5/19
Existing national and international guidelines for specific technical procedures (e.g. hospital guidelines for preventing central line-associated bloodstream infections) can be used in HHC, but need to be adapted to the home setting when needed	90	17/19
Existing national and international guidelines for specific technical procedures (e.g. hospital guidelines for preventing central line-associated bloodstream infections) cannot be used in HHC, which requires specific guidelines	31	5/16

HAI: healthcare-associated infection; HHC: home healthcare; IPC: infection prevention and control; N: number of total participants who replied; n: number of people who selected the options *“Agree”* or *“Strongly agree”*; WHO: World Health Organization.

Respondents reached consensus (≥ 80% agreement) for all statements in the first round. Statements highlighted in grey are the measures that reached consensus in the second round. Responses of *“I don’t know”* were not included in the calculations.

## Discussion

Based on the findings of this study, the definition of HAI in HHC should contain three main components: the specific link to care, the presence of a professional healthcare provider and occurrence at least 48 hours after HHC began (Table 2). The Delphi survey respondents agreed that hand hygiene, patients’ personal hygiene, training of patients and caregivers and the presence of invasive devices are risk factors for which action is necessary and feasible (Table 3). They also agreed that existing guidelines for IPC in hospital settings can and should be adapted to the specific home setting (Table 4).

The lack of a broadly accepted definition of HAI in HHC is currently the main barrier to compiling and standardising the available evidence, and our work highlights that this lack, which has been previously reported in the US [37], is also experienced by individuals working in the field in Belgium. In our opinion, this definition gap could be filled if one of the previously used definitions [20,21], together with our study results, was endorsed by an international institution such as the WHO.

Most of the information regarding risk factors for developing HAI comes from studies conducted in hospital settings, and the few studies done in homes are not consistent enough to establish robust and reproducible evidence. Previous efforts [23] and our review were not successful in identifying conclusive risk factors in the literature. Equally, our interviews highlighted an inconsistent level of awareness of risk factors for HAI in HHC among individuals working in the field. Nevertheless, most of them were aware of the general WHO guidelines on hand hygiene [38] and recognised their importance in IPC. We reached agreement on four main potential risk factors, which need to be further investigated by a study with the appropriate design and sample size.

Due to limitations in definition and risk factors, international recommendations for best practices are missing. Most published recommendations focus on ideas to initiate surveillance systems measuring infection and AE occurring in HHC, as well as to standardise indicators so that benchmarking is possible and best practices can be highlighted and implemented [39-43]. Recently, the Dutch National Institute of Public Health and the Environment published some practical and pragmatic guidelines on hygiene in home care in order to prevent HAI [44]. Given the difficulties of having better studies in the short term, we think that the empirical adaptation of hospital guidelines for IPC would be useful to all the healthcare staff working in HHC, as well as family caregivers.

A few limitations might have impacted the results of our study. First, we did not perform a systematic review of the literature, as the scoping approach allowed more flexibility in the selection process and in the evaluation of the quality of the selected publications. Second, we excluded publications before the year 2000, which we found to be outdated in regards to our research topic, and were more often discursive rather than observational or experimental studies (data not shown). Third, selection bias might have been introduced in the in-depth interviews and the Delphi survey, as participation was on a voluntary basis and might not be representative of all healthcare professionals working with HAI in HHC. Further, some participants were known and selected by two of the study researchers (ED and LM) who work in HAI surveillance in Belgian; however, we considered their network to be quite representative of the HHC professionals working in the area. More specifically, we lacked participation of academics. Finally, the number of participants in the interviews and the Delphi survey was relatively limited. However, the interviews were conducted until we reached saturation of information, and the voluntary participation in the first round of the Delphi survey was around 50% and stayed almost stable in the second round.

To our knowledge, this is the first study that attempts to disentangle various aspects of HAI in HHC in Belgium. We tackled the issue from different perspectives (literature review, qualitative interviews and quantitative survey) in order to compile a wide range of information and we strongly believe that it serves as valid ground for harmonised work and further research in order to guarantee quality across the continuum of healthcare.

To better understand the dynamics of HAI in HHC in Belgium, and to address the knowledge gaps identified, it appears necessary to conduct a national PPS that covers the whole country and uses the proposed definition. We suggest that it be conducted as an independent investigation, coordinated by a study group who could refer to the ECDC protocols of HAI in acute care hospitals [45] and HAI in European long-term care facilities [46], as well as to the French PPS on HAI in HHC reported by Miliani et al. [21].

Additionally, we suggest the development of specific recommendations and guidelines regarding the prevention and control of HAI in HHC at the national level. The drafting of these guidelines could be coordinated and written by the Belgian Superior Health Council, who could use existing national and international guidelines (e.g. WHO guidelines on hand hygiene) and empirically adapt these to the HHC context.

Our study refers mainly to Belgium, but we are aware that several HHC realities across Europe are similar to what we observed. The study recommendations—for a standardised definition of HAI in HHC and adaptation of ICP guidelines—can therefore be applicable to other countries as well. In order to harmonise definitions and practices surrounding HAI in HHC and support individual countries in dealing with the complexity of this topic, institutions such as ECDC could contribute by promoting collaboration towards a standardised definition and agreement on common ICP guidelines to use across Europe.

## Supplementary Data

960000 Supplements I–VI
