# Supplementary material for: Healthcare-associated infections in home healthcare: an extensive assessment, 2019
Source: Euro Surveill. 2021 Feb 4;26(5):1900646. doi: 10.2807/1560-7917.ES.2021.26.5.1900646 (PMC7863228; doi:10.2807/1560-7917.ES.2021.26.5.1900646)
Supplement: Supplements I–VI [file 1900646_Supplements.pdf]

Discaimer

This supplementary material is hosted by Eurosurveillance as supporting information alongside the article "Healthcare associated infections in home healthcare: an extensive assessment", on behalf of the authors, who remain responsible for the accuracy and appropriateness of the content. The same standards for ethics, copyright, attributions and permissions as for the article apply. Supplements are not edited by Eurosurveillance and the journal is not responsible for the maintenance of any links or email addresses provided therein.

List of Supplements

SUPPLEMENT I **2**

LITERATURE REVIEW STRATEGY 2

SUPPLEMENT II **3**

PROFILE AND CRITERIA OF KEY INFORMANTS INTERVIEWED AS PART OF THE HAI IN HHC STUDY 3

SUPPLEMENT III **4**

HAI IN HHC IN-DEPTH INTERVIEW GUIDE 4

SUPPLEMENT IV **6**

DELPHI QUESTIONNAIRE: FIRST ROUND 6
DELPHI QUESTIONNAIRE: SECOND ROUND 13

SUPPLEMENT V **18**

RESULTS OF THE LITERATURE REVIEW 18

SUPPLEMENT VI **28**

PARTICIPANT'S CHARACTERISTICS OF HAI IN HHC IN-DEPTH INTERVIEWS 28

Supplement I

Literature review strategy

Search terms

The two following key concepts were used for the search: both in text words (in title: abstract or key words) and subject headings when possible: *healthcare-associated infections* and *home care*. Search terms for the first concept included 'infection': 'sepsis': 'pneumonia': 'nosocomial': 'iatrogenic': 'infectious disease' and 'communicable disease': while for the second concept we used 'home care': 'home healthcare': 'home infusion': 'home based hospital care': 'hospital at home' and 'aftercare' These terms and the subjects headings were used in different combinations using the Boolean operators 'AND' and 'OR' on the selected databases.

Strings researched in each database

Pubmed

(("home care"[MeSH Terms] OR "home$care"[Title/Abstract] OR "home health$care"[Title/Abstract] OR "home infusion"[MeSH Terms] OR "home infusion"[Title/Abstract] OR "after care" [MeSH Terms] OR "after$care"[Title/Abstract] OR "home based hospital care"[Title/Abstract] OR " hospital at home"[Title/Abstract])) AND (("infection*"[Title/Abstract] OR "sepsis"[MeSH Terms] OR "sepsis"[Title/Abstract] OR "pneumonia*"[MeSH Terms] OR "pneumonia*"[Title/Abstract] OR "infectious disease*"[Title/Abstract] OR "communicable disease*"[Title/Abstract] OR "nosocomial" [Title/Abstract] OR "iatrogenic" [Title/Abstract]))

Cochrane

("home care"):ti: ab: kw AND infection:ti: ab: kw

ScienceDirect

("home care" OR "home health care" OR aftercare) AND (infection OR sepsis OR pneumonia OR "infectious disease" OR nosocomial)

CINAHL

("home care" OR "home health care" OR aftercare OR "home infusion" OR "home based hospital care" OR "hospital at home") AND (infection OR sepsis OR pneumonia OR "infectious disease" OR nosocomial OR "communicable disease" OR iatrogenic)

Embase

('home healthcare':kw: ab: ti OR 'home infusion':kw: ab: ti OR 'home based hospital care':kw: ab: ti OR 'hospital at home':kw: ab: ti OR 'home care':kw: ab: ti OR 'aftercare':kw: ab: ti) AND (infection*:ab: kw: ti OR sepsis:ab: kw: ti OR pneumonia*:ab: kw: ti OR 'infectious disease*':ab: kw: ti OR 'communicable disease*':ab: kw: ti OR iatrogenic:ab: kw: ti OR nosocomial:ab: kw: ti)

Google scholar

allintitle: ("home care" OR "home healthcare" OR "home health care" OR aftercare OR "home infusion" OR "home based hospital care" OR "hospital at home" OR "home care") infection

Supplement II

Profile and criteria of key informants interviewed as part of the HAI in HHC study

Function/ job title	Additional requirement/ criteria
1. **Nurse** conducting HHC visits	• Working as employee for an organisation specialised in providing HHC (e.g. *wit-gele kruis*)
2. **Nurse** conducting HHC visits	• Working as self-employed nurse
3. **Physiotherapist** conducting HHC visits	
4. Person providing family/household care (*familiale zorg*) to sick persons at home?	• Person should be working as employee for an organisation providing family/household care to sick persons at home
5. **General practitioner** conducting HHC visits	
6. **Patients** representative	• Member of a recognised Belgian patient platform
7. Healthcare professional involved in a **hospitalisation at home pilot projects**	• And conducting HHC visits as part of this project<br>• This might be a medical doctor, nurse or other healthcare professional
8. **Member of the management** of an organisation organising nurse HHC who is responsible for organisation and guiding of the medical aspects of this care	• Should be someone with a basic medical training (nurse, medical doctor, master in public health,…)
9. **Academic healthcare professional (meaning being part of a higher education research group)** involved in infection prevention and control	• Preferably with experiences regarding infection prevention and control in HHC and/or primary healthcare
10. **Public health manager** part of a Belgian organisation (e.g. Belgian Superior Health Council) supporting the national/regional health policies and involved in infection prevention and control	• Preferably with experiences regarding infection prevention and control in HHC and/or primary healthcare
11. **Microbiologist** involved in infection prevention and control or infectiologist	

Supplement III

HAI in HHC in-depth interview guide

1. General background

(Information on sex: place and function/job are available with the interviewer and should not be asked separately but mentioned in the minutes of the interview)

- How are you involved in advising on and/or organising and/or conducting HHC/infection prevention?
    - And since how long/ since when?

2. How would you describe/define an home care-associated infections?

- Do you use in your work already a definition for HAI in HCC and if so what is the definition used?
    - Where is this definition coming from?
    - Who/ which organisation/institution developed this definition? (APIC: ECDC: …)

- What clinical definition would you use? What epidemiological definition would you use?
- What about the fact that the HAI could be originated from a visit of the patient to an healthcare setting outside his/her home (e.g. during visit at general practitioner: during visit at specialist consultation (outpatient department) in hospital)?
    - How to consider this in the definition?

- How to report HAI in HHC?
    - Need for reporting at national level in a standardised way?
    - PPS: surveillance?
    - What to use as denominator (number patients: number device days: device insertions…?

3. What about specific risk factors for HAIs acquisition in the home care setting?

- Would you consider these risk factors different than in other settings (e.g. hospital: general practitioner practice)?
    - If so: can you explain this a bit more?
    - What are according to you the biggest differences in risk factor(s) comparing HHC and hospital care?

- What are according to you the most challenging risk factors in HHC?
- What about risk factors in the context of different kind/level of HHC - heterogeneous patient population (e.g. elderly person that needs assistants to get washed and dressed versus patient the is on IV antibiotic treatment through a peripheral catheter)?
- What about different risk factors depending of different home environment (pets: socio-economic background: home hygiene: education level patient and care-givers etc.). 'Home is designed for living and not for healthcare services'
- How to consider/manage these risk factors?
    - Only focus on those with HHC patients with increased/high risks?
    - HHC patients very heterogeneous…one-size-fits-all approach not possible in management of risk factors and infection prevention.
- What is according to you the risk to develop an HAI in HHC? Higher than in healthcare facility (acute/long-term health facility)?

4. What about prevention and control of home care-associated infections in Belgium?

- How to prevent and control HAI in HHC?
- What is needed to prevent and control HAI in HCC? (training: communication between different healthcare settings – continuum of care: recording and reporting: definitions and priorities at national level:)
- Do you know about guidelines on how to prevent and control HAI in HCC?
  - Who developed these guidelines? Where are these guidelines coming from?
  - Are these guidelines used/implemented in HHC in Belgium? If so: where? By whom?
  - Do you have comments on these guidelines? If so: what kind?
  - Are these guidelines according to you useful for the HHC setting or should there be changes in these guidelines? If so: what kind of changes?
- How would you deal with specific HHC HAI risk factors?
- How would you deal with different kind/level of care provided in HHC (e.g. elderly person that needs assistants to get washed and dressed versus patient the is on IV antibiotic treatment through a peripheral catheter).
- What about different risk factors depending of different home environment (pets: socio-economic background: home hygiene: etc.)? How to manage this?
- Do you know about guidelines and interventions already implemented in Belgium by healthcare workers providing HHC to prevent and control HAI?
  - If so: what by whom and what are the experiences (outcome) with the implementation of these guidelines and interventions?

Supplement IV

Delphi questionnaire: first round

Introduction

Dear participant:

Thank you for taking part in the 'Healthcare-Associated Infections (HAI) in Home Healthcare (HHC)' Delphi survey. The survey is part of a broader project which aims to: formulate a definition of HAI in HHC: identify their specific risk factors: and develop a standardized framework for prevention and control of HAI in HHC in Belgium.

We have so far performed a literature review and conducted several in-depth interviews: which were used to design this two-steps Delphi survey. Its aim is to reach a consensus between experts on the main results.

In this first round: you will be asked to provide some information about your background and profession: and to answer questions related to five main areas:

1. *Definitions of HAI in HHC;*
2. *Reporting of HAI in HHC*
3. *Risk factors for HAI in HHC;*
4. *Measures and guidelines to prevent and control HAI in HHC.*

In total the survey should take between 25 and 35 minutes. We are eager to learn about your opinions and thank you for your collaboration and your time!
Please click 'Next' at the bottom of the screen to proceed

This project has been initiated and funded by the King Boudouin Foundation and is being carried out by Sciensano: the Belgian Federal Public Health Institute.

Participation in this survey is voluntary and completely anonymous.

Participant's information

In this section: we would like to have more information about your background: relevant to Healthcare-Associated Infections (HAI) in Home Healthcare (HHC).

1. **How many years of working experience do you have in healthcare?** _____

2. Please indicate how much you are involved in the following professional activities:
   - providing home healthcare
   - Involved in policy making
   - Involved in the management of an organization offering home healthcare services
   - Academic healthcare professional
   - Involved in a hospital at home (eg/OPAT) project
   - Involved in infection prevention and control
   - Public Health management

   Scale: never-often

3. If you have experience in home healthcare: please indicate how **many years of working experience you have in this field (if no experience: indicate 0)**

Definitions of HAIs in HHC

In this section: we aim to investigate if there is information: need for and agreement on definition of Healthcare-Associated Infections (HAI) in Home Healthcare (HHC).

4. **Do you know a definition of HAI in HHC?**
   - ☐ Yes
   - ☐ No

   IF Yes: Could you write the definitions you know: or cite the source who defines it?
   ____

   IF No: Go to question 5 directly

5. **Do you agree there should be a standardized definition of HAI in HHC?**
   - ☐ Yes
   - ☐ No

   IF Yes: Go to question 6 directly

   IF No: Could you please motivate your answer?

   ____

6. **Given the following definitions of HAI in HHC: which we encountered during our literature review and in-depth interviews: could you please say how much you agree with each of them?**

**Nr.**	***List of Definitions***	**Strongly disagree**	**Disagree**	**Agree**	**Strongly agree**	**I don't know**
6.1	Any infection that develops in a patient who is cared for at home	○	○	○	○	○
6.2	Any infection that develops in a patient who is cared for at home: and is related to receiving healthcare	○	○	○	○	○
6.3	Any infection that is associated with a medical or surgical intervention and that was neither present nor incubating at the time of initiation of care in the patient's home	○	○	○	○	○
6.4	Any infection that develops in a patient who receives HHC from a professional healthcare worker and that occurs 48 hours or later after initiating this HHC	○	○	○	○	○
6.5	Any infection that can be specifically linked with providing care (e.g. wound infection: infection linked with the use of catheters) that develops in a patient who receives HHC from a professional healthcare worker	○	○	○	○	○
6.6	Any infection that can be specifically linked with providing care (e.g. wound infection: infection linked with the use of catheters) that develops in a patient who receives HHC from a professional healthcare worker and that occurs 48 hours or later after initiating this HHC	○	○	○	○	○

If you would like to suggest another definition of HAI in HHC in Belgium: please do so here (optional): ____

Reporting of HAIs in HHC

In this section: we would like to know your views on the importance of Healthcare-Associated Infections (HAI) in Home Healthcare (HHC): whether this problem requires a reporting and if so: by which method.

7. How much do you agree with the following statements regarding the prevalence of HAI in HHC in Belgium?

Nr.	*Prevalence of HAI in HHC in Belgium is…*	Strongly disagree	Disagree	Agree	Strongly agree	I don't know
7.1	Most probably low	O	O	O	O	O
7.2	Not known: but lower than in hospitals and in nursing homes	O	O	O	O	O
7.3	Not known: and doesn't need to be measured because at present HAI in HHC is not a priority healthcare issue	O	O	O	O	O
7.4	Not known: but needs to be measured (at least once) to know the situation regarding HAI in HHC	O	O	O	O	O
7.5	Underestimated and minimalized because data is lacking	O	O	O	O	O
7.6	Most probably high	O	O	O	O	O

If you have another opinion regarding the prevalence of HAI in HHC in Belgium: please state it here (optional):

8. How much do you agree with the following statements regarding the need to report HAI in HHC in Belgium?

Nr.	*Statement*	Strongly disagree	Disagree	Agree	Strongly agree	I don't know
8.1	Reporting of HAI in HHC is currently not needed since it is not a healthcare priority	O	O	O	O	O
8.2	Reporting of HAI in HHC is needed for all occurring infections	O	O	O	O	O
8.3	Reporting of HAI in HHC is needed because at present there is no data available which makes it difficult to know its importance; it might be overestimated or underestimated	O	O	O	O	O
8.4	Reporting of HAI in HHC is needed only for those infections that can be specifically linked with providing care (e.g. wound infection: infection linked with the use of catheters) in HHC	O	O	O	O	O
8.5	Reporting of HAI in HHC should be based on diagnostic microbiological elements and not only on infections' signs and symptoms	O	O	O	O	O

If you have another opinion regarding the need to report HAI in HHC in Belgium: please state it here (optional) : ____

9. **How much do you agree with the following statements regarding <u>ways</u> to report HAI in HHC in Belgium?**

Point Prevalence Survey (PPS): study done on a given population which measures the prevalence (proportion of cases) of a defined problem at a specific point in time.

Surveillance system: continuous: systematic data collection and analysis.

**Nr.**	***There is need for....***	**Strongly disagree**	**Disagree**	**Agree**	**Strongly agree**	**I don't know**
9.1	A study to quantify the prevalence of different HAI and obtain information on the importance of the issue	O	O	O	O	O
9.2	A specific study on MRSA (and other MDRO) prevalence in HHC patients and their caregivers (including nurse: GP: etc.)	O	O	O	O	O
9.3	A point prevalence survey (PPS): organized only once: that includes all HAI occurring in HHC	O	O	O	O	O
9.4	A PPS organized only once including only HAI that can be specifically linked with providing care (e.g. wound infection: infection linked with the use of catheters) occurring in HHC	O	O	O	O	O
9.5	A PPS that is repeated on regular basis (e.g. every 3 to 5 years)	O	O	O	O	O
9.6	A surveillance system including all HAI occurring in HHC	O	O	O	O	O
9.7	A surveillance system including only HAI that can be specifically linked with providing care (e.g. wound infection: infection linked with the use of catheters) occurring in HHC	O	O	O	O	O
9.8	Start with a PPS and depending on the results of the PPS set up a surveillance system for some type of HAI	O	O	O	O	O

If you would like to suggest another way to report HAI in HHC in Belgium: please do so here (optional): ____

Risk Factors for HAIs in HHC

In this section: we seek to identify the specific risk factors that contribute to the acquisition of Healthcare-associated infections (HAI) in the home setting: as compared to the hospital setting.

10. **How much do you agree with the following statements regarding risk factors for HAI in HHC?**

**Nr.**	***Statement***	**Strongly disagree**	**Disagree**	**Agree**	**Strongly agree**	**I don't know**
10.1	Risk factors for HAIs in home care are different than those in hospital care	O	O	O	O	O
10.2	The risk for HAIs is higher in hospital care than in home care	O	O	O	O	O
10.3	There is a need for additional research on identification of the risk factors for HAIs in HHC	O	O	O	O	O

11. **Given the following risk factors: could you please report what you think is their importance in developing a HAI in HHC?**

Nr.	*List of Risk Factors*	Very low	Low	Intermediate	High	Very high	I don't know
Risk factors linked with patient's lifestyle and socio-economic status							
11.1	Patient's personal hygiene	○	○	○	○	○	○
11.2	Home hygiene	○	○	○	○	○	○
11.3	Home infrastructure (presence of sanitations: soap…)	○	○	○	○	○	○
11.4	Presence of pet in the home environment	○	○	○	○	○	○
11.5	Education level of the patient	○	○	○	○	○	○
11.6	Presence of caregiver(s) in the household	○	○	○	○	○	○
11.7	Socio-economic status of the patient	○	○	○	○	○	○
11.8	Training of patient and caregiver(s) about the measures to prevent HAI in HHC	○	○	○	○	○	○
Risk factors linked with patient's characteristics and pathology							
11.9	Patient's age	○	○	○	○	○	○
11.11	Patient's gender	○	○	○	○	○	○
11.11	Underlying health condition of the patient	○	○	○	○	○	○
11.12	Medical condition for which HHC was indicated	○	○	○	○	○	○
11.13	Presence of invasive devices	○	○	○	○	○	○
11.14	Duration of the presence of invasive devices	○	○	○	○	○	○
11.15	Duration of the home care	○	○	○	○	○	○
Risk factors linked to care							
11.16	Hand hygiene of healthcare provider	○	○	○	○	○	○
11.17	Management of invasive devices by the healthcare provider	○	○	○	○	○	○
11.18	Frequency of visits by healthcare provider	○	○	○	○	○	○
11.19	Lack of time by the healthcare provider during the visit	○	○	○	○	○	○
11.20	Communication between different care providers	○	○	○	○	○	○

If you would like to suggest other risk factors of HAI in HHC: please do so here (optional):

____

Measures and guidelines to prevent and control HAIs in HHC

In this section: we seek to investigate which measures would be most appropriate to prevent and control Healthcare-Associated Infections (HAI): as well as the need for specific guidelines in the home setting: as compared to the hospital setting.

12. How much do you agree with the following statements regarding <u>measures</u> to prevent and control HAI in HHC in Belgium?

By measures: we mean plans or actions taken to achieve a particular purpose (protect patients: visitors and staff from acquiring an infection such as appropriate environment hygiene or waste management).

Nr.		Strongly disagree	Disagree	Neutral	Agree	Strongly agree	I don't know
12.1	Infection prevention and control (IPC) measures of hospital care should be applied to home care	O	O	O	O	O	O
12.2	IPC measures in HHC should not be as strict as in hospital care	O	O	O	O	O	O
12.3	IPC measures in HHC should be based on the health condition of the patients receiving care and the type of care provided	O	O	O	O	O	O
12.4	There is a need for specific procedures for the management of invasive devices in HHC	O	O	O	O	O	O
12.5	If needed: procedures for the management of invasive devices in hospital can be applied to HHC	O	O	O	O	O	O
12.6	The implementation of IPC measures is a shared responsibility between the healthcare providers: the family caregivers and the patients themselves	O	O	O	O	O	O
12.7	Training for patients and caregivers is essential in the implementation of IPC measures.	O	O	O	O	O	O
12.8	Monitoring of the implementation of IPC measures in HHC is needed	O	O	O	O	O	O
12.9	Better communication between hospitals: GPs and HHC nurses is needed	O	O	O	O	O	O
12.10	Patient empowerment is needed and key for good IPC in HHC	O	O	O	O	O	O
12.12	There should be more pre-service training on IPC of HAI in HHC in medical and nursing schools in Belgium	O	O	O	O	O	O
12.12	There should be more post-graduate training possibilities on IPC of HAI in HHC for healthcare workers providing HHC in Belgium	O	O	O	O	O	O
12.13	In IPC management in HHC it is important to differentiate between standardized measures that apply to all patients and additional measures that apply to specific patients and care (e.g. immunocompromised patients: sterile wound infection)	O	O	O	O	O	O

If you would like to suggest other measures to prevent and control HAI in HHC in Belgium: please do so here (optional):

____

13. How much do you agree with the following statements regarding <u>availability and need for guidelines</u> to prevent and control HAI in HHC in Belgium?.

By guidelines: we mean formalized documentation recommending how something should be done such as guideline for prevention of catheter-associated urinary tract infections.

**Nr.**	***Statements***	**Strongly disagree**	**Disagree**	**Neutral**	**Agree**	**Strongly agree**	**I don't know**
13.1	There are no specific guidelines to prevent and control HAI in HHC for Belgium	O	O	O	O	O	O
13.2	Existing guidelines for hospital setting can be adapted and used in the home care setting	O	O	O	O	O	O
13.3	There is a need for standardized IPC guidelines for HHC but these must leave room and possibilities for adaptation to the local home and HHC context	O	O	O	O	O	O
13.4	There is a need for standardized general guidelines that apply to all patients receiving HHC	O	O	O	O	O	O
13.5	There is a need for standardized specific guidelines that apply to patients receiving more complex HHC	O	O	O	O	O	O
13.6	It would be an added value if national standardized guidelines on IPC of HAI in HHC would be available and accessible to all the staff involved in delivering HHC	O	O	O	O	O	O
13.7	IPC guidelines related to specific technical procedures provided in HHC (e.g. hospital at home: outpatient parenteral antibiotic therapy) should be standardized nationally and not differ according to the referring hospital	O	O	O	O	O	O
13.8	There is an urgent need for IPC guidelines specific for HHC	O	O	O	O	O	O

If you have another opinion regarding the need and availability of guidelines to prevent and control HAI in HHC in Belgium: please state it here (optional):

____

Delphi questionnaire: second round

Introduction

Dear participant:

Thank you for taking part in this second round of the Delphi Survey on 'Healthcare-Associated Infections (HAI) in Home Healthcare (HHC)'. As you know: the survey is part of a broader project which aims to: formulate a definition of HAI in HHC: identify their specific risk factors: and develop a standardized framework for prevention and control of HAI in HHC in Belgium.

We have so far performed a literature review: conducted several in-depth interviews and completed the first Delphi round: whose results were used to design this second round. The Delphi method aims to reach a consensus between experts on relevant topics.

In this second round: you will find preliminary results of the first round: and will be asked to provide some more information on topics for which there was still ambiguity. We also added a few questions to investigate further some topics or to add input from the comments received during the first round. The four main areas investigated are:

1. *Definition of HAI in HHC;*
2. *Reporting of HAI in HHC*
3. *Risk factors for HAI in HHC;*
4. *Measures and guidelines to prevent and control HAI in HHC.*

In total the survey should take ***no more than 15 minutes****. We are eager to learn about your opinions and thank you for your collaboration and your time!*
Please click 'Next' at the bottom of the screen to proceed

This project has been initiated and funded by the King Boudouin Foundation and is being carried out by Sciensano: the Belgian Federal Public Health Institute.

Participation in this survey is voluntary and completely anonymous.

Participant's information

n this section: we would like to have more information about your background: relevant to Healthcare-Associated Infections (HAI) in Home Healthcare (HHC).

**Q1**	How many years of working experience do you have in **healthcare**?	
**Q2**	If you have experience in **home healthcare**: please indicate how many years of working experience you have in this field (if no experience: indicate 0)	

Q3. Please indicate how much you are involved in the following professional activities:

Nr.		**never**	**rarely**	**sometimes**	**often**
3.1	Providing home healthcare	O	O	O	O
3.2	Involved in policy making	O	O	O	O
3.3	Involved in the management of an organization offering home healthcare services	O	O	O	O
3.4	Academic healthcare professional	O	O	O	O
3.5	Involved in a hospital at home (eg/OPAT) project	O	O	O	O
3.6	Involved in infection prevention and control (IPC)	O	O	O	O
3.7	Public Health management	O	O	O	O

OPAT: Outpatient parenteral antimicrobial therapy

Definitions of HAIs in HHC

In the first Delphi round: all respondents agreed there should be a standardized definition of Healthcare-Associated Infections (HAI) in Home Healthcare (HHC).

Q4. Among the suggestions made: two statements obtained a level of agreement of more than 85%. Could you please select the statement you most agree with?

Nr.	***Definitions***	Agree most	Results round 1: % agreed and strongly agreed
4.1	Any infection that develops in a patient who receives HHC from a professional healthcare worker and that occurs 48 hours or later after initiating this HHC	○	86
4.2	Any infection that can be specifically linked with providing care (e.g. wound infection: infection linked with the use of catheters) that develops in a patient who receives HHC from a professional healthcare worker and that occurs 48 hours or later after initiating this HHC	○	90

If you have a comment: please state it here (optional):

____

Reporting of HAIs in HHC

In the first Delphi round: all respondents agreed reporting was needed in order to get an idea of the prevalence of the problem. More than 80% of them believed it was currently underestimated.

Q5. Among the suggestions made regarding the **need to report HAI in HHC in Belgium**: two statements did not reach a high level of agreement. Could you please select the statement you most agree with?

Nr.	***Statement***	Agree Most	Results round 1: % agreed and strongly agreed
5.1	Reporting of HAI in HHC is needed for all occurring infections	○	45
5.2	Reporting of HAI in HHC is needed only for those infections that can be specifically linked with providing care (e.g. wound infection: infection linked with the use of catheters) in HHC	○	75

If you have a comment: : please state it here (optional):

____

In the first Delphi round: 90% of the respondents agreed to start with a point prevalence survey (PPS) and depending on the PPS result: set up a surveillance system. More than 80% of the respondents also agreed to repeat a PPS on a regular basis (every 3 to 5 years).

Q6. Among the suggestions made regarding the **ways to report HAI in HHC in Belgium**: two statements did not reach a high level of agreement. Could you please select the statement you most agree with?

Point Prevalence Survey (PPS): study done on a given population which measures the prevalence (proportion of cases) of a defined problem at a specific point in time.

Surveillance system: continuous: systematic data collection and analysis.

Nr.	*There is need for….*	Agree most	Results round 1: % agreed and strongly agreed
6.1	A point prevalence survey (PPS) that includes all HAI occurring in HHC	O	57
6.2	A PPS including only HAI that can be specifically linked with providing care (e.g. wound infection: infection linked with the use of catheters) occurring in HHC	O	56

Q7. Among the suggestions made regarding the **ways to report HAI in HHC in Belgium**: some statements did not reach a high level of agreement and needed to be investigated further. We therefore added or rephrased some questions for more clarification. Could you please say how much you agree with each of the following statements?

Nr.		Strongly disagree	Disagree	Agree	Strongly agree	Results round 1: % agreed and strongly agreed
7.1	Reporting of HAI in HHC should be based on diagnostic microbiological elements and not only on infections' signs and symptoms	O	O	O	O	74
7.2	There is need for a specific study on MRSA (and other MDRO) prevalence in HHC patients and their caregivers (including nurse: GP: etc.)	O	O	O	O	65
7.3	Prevalence of MRSA (and other MDRO) in HHC **patients and their caregivers** should be included in the PPS	O	O	O	O	NA*
7.4	Prevalence of MRSA (and other MDRO) in HHC **patients only** (and not the caregivers) should be included in the PPS	O	O	O	O	NA*

** 'NA': not applicable: as this question was not asked in the first round*

If you have a comment: please state it here (optional):

____

Risk Factors for HAIs in HHC

In the first Delphi round: all respondents agreed there was a need for additional research on identification of risk factors for HAIs in HHC.

Q8. For the following risk factors for HAI in HHC: a level of agreement higher than 80% of the respondents was reached. Could you please select the five risk factors you believe are **most needed to act on** and the five risk factors you believe are **most feasible to act on?**

Nr.	*List of Risk Factors*	Action most needed	Action most feasible	Results round 1: % agreed and strongly agreed
8.1	Patient's personal hygiene	○	○	100
8.2	Home infrastructure (presence of sanitations: soap…)	○	○	100
8.3	Socio-economic status of the patient	○	○	84
8.4	Training of patient and caregiver(s) about the measures to prevent HAI in HHC	○	○	95
8.5	Patient's age	○	○	81
8.6	Underlying health condition of the patient	○	○	100
8.7	Medical condition for which HHC was indicated	○	○	95
8.8	Presence of invasive devices	○	○	100
8.9	Duration of the presence of invasive devices	○	○	100
8.10	Duration of the home care	○	○	81
8.11	Hand hygiene of healthcare providers	○	○	95
8.12	Management of invasive devices by healthcare providers	○	○	100
8.13	Frequency of visits by healthcare providers	○	○	90
8.14	Lack of time by the healthcare providers during the visit	○	○	80

If you have a comment: please state it here (optional):

____

Measures and guidelines to prevent and control HAIs in HHC

In the first Delphi round: nearly all the statements regarding **measures and guidelines to prevent and control HAI in HHC** in Belgium obtained a level of agreement higher than 80%.

By measures: we mean plans or actions taken to achieve a particular purpose (protect patients: visitors and staff from acquiring an infection such as appropriate environment hygiene or waste management).

By guidelines: we mean formalized documentation recommending how something should be done such as guideline for prevention of catheter-associated urinary tract infections

Q9. Among the suggestions regarding **measures and guidelines to prevent and control HAI in HHC** in Belgium: some statements did not reach a high level of agreement and needed to be investigated further. We therefore added or rephrased some questions for more clarification. Could you please say how much you agree with each of the following statements?

Nr.	*Statements*	Strongly disagree	Disagre e	Agree	Strongl y agree	Results round 1: % agreed and

						strongly agreed
9.1	Existing national and international accepted infection prevention and control (IPC) guidelines for HAI (e.g. WHO guidelines on hand hygiene) can be used in HHC without adaptation	O	O	O	O	NA*
9.2	Existing national and international accepted IPC guidelines for HAI (e.g. WHO guidelines on hand hygiene) can be used in HHC but need to be adapted to the home setting when needed	O	O	O	O	NA*
9.3	Existing national and international accepted IPC guidelines for HAI (e.g. WHO guidelines on hand hygiene) cannot be used in HHC: which requires specific guidelines	O	O	O	O	NA*
9.4	Existing national and international guidelines for specific technical procedures (eg/ hospital guidelines for preventing central line-associated bloodstream infection) can be used in HHC without adaptation	O	O	O	O	NA*
9.5	Existing national and international guidelines for specific technical procedures (eg/ hospital guidelines for preventing central line-associated bloodstream infections) can be used in HHC but need to be adapted to the home setting when needed	O	O	O	O	NA*
9.6	Existing national and international guidelines for specific technical procedures (eg/ hospital guidelines for preventing central line-associated bloodstream infections) cannot be used in HHC: which requires specific guidelines	O	O	O	O	NA*

** 'NA': not applicable: as this question was not asked in the first round*

If you have a comment: please state it here (optional):

____

Supplement V

Results of the Literature review

Study information							Type of HAI	Contents - Findings		
Nr	First Author: year		Study type	Setting	Population	Aims		Definitions of HAI in HHC	Risk factors of HAI in HHC	Recommendations for prevention and control
1	Al Wakeel: 2018	Comment	Retrospective analysis	Saudi Arabia	>16 years	Complications and outcomes in PD in home	Peritonitis ESI	no info	Assisted peritoneal dialysis was comparable with self care peritoneal dialysis	Implementation of protocols can reduce the risks of infections and other dialysis-related complications
		Score	++	+	+++		+++	-	+++	++
2	Barr: 2013	Comment	Expert overview	UK	no info	To outline good practice recommendations and highlight OPAT management of infections	device related	no info	- indwelling intravascular access devices<br>- HAI rates are lower than in hospital	To record OPAT adverse outcomes for quality assurance purposes
		Score	+	++	-		+++	+++	++	++
3	Bech: 2016	Comment	Observational cohort	Denmark	all age groups	To investigate whether environment risk factors influence the time of 1st CRBSI	CRBSI	CRBSI was defined as no other obvious infection sites and a positive blood culture both peripherally and from the catheter	- PICC increased risk when compared with Hickman catheter<br>- Hickman catheter had increased risk if managed by a nurse	- Revision of the current allocation guidelines<br>- PICCs should be used only for short- term HPN therapy and when few infusion days per week are needed<br>- Management of the Hickman catheters by home care nurses should be further specialized
		Score	++	++	+++		+++	+++	+++	++++
4	Blais: 2013	Comment	Retrospective cohort study	Canada	all age groups	To document the incidence rate and types of adverse events (AEs) among HC clients and identify factors contributing to these AE	Infection/ sepsis	Patients who acuired an infection during their period of home care	- Acquiring an infection/sepsis was associated with the presence of an AE	- Homes are not designed healthcare and ar enot regulated envionments<br>- Consider role of unpaid caregivers when planning strategies to improve HC safely
		Score	++	+	+++		++	-	+	+++
5	Buchman: 2013	Comment	Retrospective chart review	USA	all age groups	To determine CRBSI risk factors in HPN	CRBSI	Clinical symptoms consistent with systemic infection while infusing PN; fatigue; weight loss; the absence or pres- ence of blood leukocytosis: together with positive blood cultures obtained via the CVC and peripherally and from the catheter tip (when the catheter required removal); and the absence of other potential sources of infection	- Being adult compare to being child<br>- Lipid infusion (in adults)<br>- Blood drawing from the CVC<br>- Frequency of infusions (children)<br>- Catheter type (infusion ports>tunneled catheter)<br>- Number of lumens (1<2<3)<br>- Non-PN catheter use	Patients in long-term PN should have single-lumen: tunneled catheter which is used exclusively for PN infusion. Blood samples should not be obtained from the PN catheters: and number of non-PN infusions should be limited. Consider decreasing the lipid emulsion infusion: at least in adults.
		Score	++	+	+++		+++	+++	+++	+++
6	Chenoweth: 2007	Comment	Retrospective cohort study	USA	all age groups	To identify demographic characteristics of patients: risk factors and outcomes associated with the development of VAP in home care	VAP	Infection in patients who received home mechanical ventilation and were followed up by respiratory therapists	- Longer duration of the ventilation	Interventions may be directed at the education and support of caregivers and focused toward those patients requiring longer daily periods of ventilation.
		Score	++	+	+++		+++	++	+++	+++
7	Cheung: 2008	Comment	RCT	Hong-Kong	>18 years	To compare the risk of acquiring UTIs through the conventional practice of using	UTI	no info	no info	Using sterile water to clean the periurethral area before catheterization among home care

						0.05% chlorhexidine gluconate (CHG) versus sterile water for periurethral cleansing before insertion of a urinary catheter				patients will not increase the risk for urinary tract infections
		Score	+++	+	+++		+++	-	-	+++
8	Cornillon: 2017	Comment	Prospective survey	France	all age groups	To report on the systematic use of PICC for outpatient care after an allogeneic HSCT	CRBSI	The presence of the same organism grow from at least 1 percutaneous blood culture and from a culture of the catheter tip: or that 2 blood samples be drawn (one from a catheter hub and the other from a peripheral vein) that: when cultured: meet CRBSI criteria for quantitative blood cultures or differential time to positivity	no info	The systematic use of the PICC in a population of outpatients returning home after an allogeneic HSCTseems feasible and safe: regarding the low rate of complication
		Score	++	++	+++		+++	++	-	++
9	Cox.: 207	Comment	Retrospective cohort study	USA	all age groups	To determine whether older adults were able to successfully administer home IV antimicrobials without intensive support from home care agencies	device related	no info	Only 3/231 BSI occurred and no specific risks was identified. The numbers are low because patients were not in PN and not immunosuppressed.	The assessment of the patient's and the caregiver's abilities to perform home infusion is crucial to the success of any home infusion program
		Score	++	+	+++		+++	-	+++	+++
10	Doran: 2014	Comment	Delphi survey	Canada	all age groups	To assess which client events should be considered reportable and preventable in home care	different HAI	A new infection related to an IV: PICC: central catheter site or related blood stream	no info	90% of exeperts agreed that HAI should be reportable; meaning making the event known to a higher authority
		Score	+	+	+++		+++	+++	-	+++
11	Doran: 2013	Comment	Retrospective cohort study	Canada	all age groups	To investigate a significant safety dimension of HC: the occurrence of adverse events and their related outcomes	different HAI	**Surgical wound infection**: infection present on any ED visit or hospital admission within 30 days of a hospitaldischarge with open surgery but without infection recorded **Ventilator-associated pneumonia**: pneumonia present on any ED visit or hospital admission within 30 days of RAI-HC assessment among clients who had ventilator documented but didn't have pneumonia recorded at the time of assessment **Catheter-associated UTI**: UTI present on any ED visit or hospital admission within 30 days of RAI-HC assessment among clients who had indwelling urinary catheter documented but didn't have UTI recorded at the time of assessment **Peripheral IV infection**: bacteremia or localized skin infection present on any ED visit or hospital admission within 60 days of RAI-HC assessment among clients who had peripheral IV infusion documented at the time of assessment **Central line IV infection**: Bacteremia or localized skin infection present on any ED visit or hospital	no info	no info

								admission within 60 days of RAI-HC assessment among clients who had central IV infusion documented at the time of assessment		
		Score	++	+	+++		+++	+++	-	-
12	Embry: 2000	Comment	Draft definitons for surveillance	USA	NA	Draft definitons for surveillance	different HAI	**Home health–associated infections** refer to infections that develop in patients who are receiving home health care and that were neither present nor incubating at the time the patient began receiving home health are (generally: 48–72 hours after admission but may vary based on the incubation time of the infecting pathogen).<br>**For specific definitions refer to the full text**	NA	NA
		Score	++++	+	-		+++	+++	-	-
13	Friedman: 2000	Comment	Expert essay	USA	NA	Overiew of infections control in home care	no info	no info: refers to some old CDC guideline (1988)	Nurse's bag<br>Devices (noncritical: semicritical: critical)	It is imperative that home care organizations begin to collect data on HAI in an organized fashion and share their results through meetings and publications
		Score	+	+	-		-			++
14	Gallone: 2016	Comment	Survey	Italy	all age groups	To assess HAI prevalence	different HAI	no info	bed sores: urinary catheter: gastrostomy: tracheostomy: vascular ulcers: diabetic ulcers: mechanical ventilation: ureterostomy: vascular catheter: and ileostomy	Need for a new framework of health system to strictly monitor the phenomenon of HAI in all settings (hospital: nursing home: long-term care facilities: and home care) through an integrated approach: because patients often move from one assistance setting to another: and microorganisms come next after patients
		Score	++	++	+++		+++	-	++	+++
15	Gorski: 2004	Comment	Survey	USA	all age groups	To investigate the outcomes of central venous access devices (CVAD) in homecare	CVAD	CVAD infection if following criteria are met in >48 from admission or after insertion of CVAD:<br>• Must have a CVAD present and the finding (by blood culture) of bacteremia **OR**<br>• CVAD present and two of the following:<br>- Temperature 2° F or ° 1 C over the patient's baseline or chills<br>- Significant local pain and redness associated with the central catheter or exit site<br>- Hypotension (90 mm Hg systolic)<br>- MD suspects central line infection is present and initiates therapy (changes/removes line or starts antibiotics)<br>- WBC count 10: 000 **OR**<br>• Pus: cellulitus: or significant pain present at exit site of central catheter	Multi-lumen catheter: Receiving TPN: Neutropenia: Immunosuppressive drugs: Prior history of CVAD infection: Poor nutrition: Impaired skin integrity: Technique failure: Showering and getting dressing wet	To reduce complications rate:<br>- Identify patients at higher risk of HAI<br>- Involve staff in data collection<br>- Monitor and educate patients<br>- Develop preventive protocols for patients at risk
		Score	++	+	+++		+++	+	+++	+++
16	Von Baum: 2010	Comment	Environmental study	Germany	all age groups	To assess whether tap water from the domestic environment	different waterborne infections	no info	no info	Although the risk of infection from household water-borne pathogens appears low: preventive measures

						of neutropenic patients poses a risk for infections from the waterborne pathogens				may be considered on an individual basis in patients with long-term immunosuppression as well as in patients with long-term central-vascular catheterization
		Score	+++	++	+++			-	-	+
17	Gorski: 2010	Comment	Expert essay	USA	NA	Review of the etiology: identification: and best practices related to central vascular access devices (CVAD)	CVAD	no info	Potential source of infection:<br>- Infusate<br>- Skin<br>- Microbial contamination at the catheter hub or needleless connector	Nurses must:<br>- know the infection transmissions<br>- apply infection prevention interventions (hand hygiene: aseptic techniques: and infusion administration)<br>- educate patients on risks and signs of infection<br>- investigate any occurance of infection to prevent it from happening again.
		Score	+	+	-		+++	-	++	+++
18	McGoldrick: 2010	Comment	Expert essay	USA	NA	Evidenced- based guidelines and recommendations on methods for managing respiratory equipment used by patients in the home setting and surveillance activities to prevent respiratory infections	Respiratory HAI	no info	Risk/Protective factors:<br>- Hand hygiene<br>- Respiratory equipment (noncritical: semicritical: critical)<br>- Oral care<br>- Equipment cleaning<br>- Patients education	Implement evidence-based practices: by organization's staff: patients and caregivers. Combine use hand hygiene: barrier precautions: and meticulous cleaning and disinfection with an EPA registered products.
		Score	+	+	-		+++	-	++	
19	Horcajada: 2007	Comment	Retrospective analysis	Spain	all age groups	To review the experience of home care service from 1995 to 2002 using prospectively recorded data	CRBSI	Sepsis of the catheter	Self administration of the parenteral treatment	Patients that selfadminister the treatments should be selected and trained appropriately: insisting on the hygiene measures and on how to heandle the venous catheter.
		Score	++	++	+++		+++	-	+++	+++
20	Shang: 2014	Comment	Systematic review	USA	all age groups	To critically review and synthesize published evidence on infection prevalence and risk factors among adult patients who received HHC services and to evaluate the methodologic quality of these studies	different HAI	no info	**IV catheter**:<br>- <u>patiets characteristic or medical history</u> (younger age: undelying disease: bone marrow trasplant: receiving TPN treatment: infusion therapy outside home: HPN<5 years: family memebr with HPN)<br>- <u>catheter related factors</u> (multilumen catheter: central venous access salvage)<br>- <u>patient's social or economic factors</u> (being part time student: recipient of social welfare)<br>**Catheter related UTI**:<br>being a woman: catheter change interval <4 weeks: number of nurses change catheters<br>**Ventilator associated pneumonia**:<br>daily duration of ventilation<br>**Non-specific infections:**	- Identify HHC patients who are at high risk for infection<br>- Give special attention to patients receiving PN because they experience the higher rates of infections<br>- 1-size-fits-all approach is not appropriate in infection control for HHC settings<br>- Assess home environment during 1st home visit<br>- Tailored educatin for the patient and caregivers<br>- Facilitate efficient and effective communication between the different actors in the transition between home care: hospitals and long term facilities.

									indewelling devices: service offered by agency which offers paid sick leave to the staff	
		Score	+++	+	+++		+++	-	+++	+++
21	Schildmeijer: 2018	Comment	Retrospective record review	Sweden	>18 years	To explore the origin: incidence: types and preventability of the AEs that occur in patients receiving home healthcare	different HAI	Deficniton was given for AE: The AE occurred during the index admission: that is: within 90 days after admission in home healthcare: regardless of caregiver.<br>I think the HAI in HHC was defined as the HAI in hospital but there is no clear definition.	no info	The study found that 64% of HAI were preventable: meaning that they could have been prevented if adequate measures and/ or actions had been taken during the patient's contact with healthcare. This definition is based on the terminology in the Swedish Patient Safety Act.
		Score	++	++	+++		+++	+	-	+
22	Keller: 2018	Comment	Expert overview	USA	NA	Needs and requirements for reporting CLABSIs in home infusion therapy	CLABSI	Mentions the APIC definition as the reference: but argues that a denomitor definition is missing and therefore the measurement is not possible for benchmarking	no info	Reports that the 21st Century Cures Act in the US will expand Medicare coverage for home infusion therapy services by 2021 and that this act will likely lead to increased pressure for home infusion therapy CLABSI surveillance
		Score	+	+			+++	+	-	+
23	Manangan: 2002	Comment	Expert overview	USA	NA	Feasibility of National surveillance of HAIs in home care setting	NA	Agrees with APIC definitions draft (Embry 2000)	no info	Lists the challenges in having the surveillance system and its usefulness in understanding the epidemiology of HAI in HHC and provide mean for M&E of interventions
		Score	+	+				+	-	+
24	Masotti: 2010	Comment	Scoping review	Canada	all age groups	To map the extent and range of existing research on the AE of home care. Six topics: definitions: rates: causes: consequences: interventions and policy	CLABSI<br>CAUTI<br>Ventilator associated infections<br>Wound infections<br>(also others)	Definition of AE: events or occurrences which become apparent during the delivery of home home care services: and which have a negative impact on patient care: patient outcomes: family or support care and resources utilization	**Patient-level characteristics**: increased age and co-morbidities: gender: depression: cognitive impairments: functional status/limitations: patient compliance and living alone or no caregiver<br>**Organization and system-level** characteristics: communication: coordination: collaboration : team experience: training or knowledge: team workload: medication errors: unrecognized polypharmacy: drug label instructions: and inadequate patient monitoring/assessment	- Have more research for home care AE<br>- The rates are most probably understimated: conside higher<br>- Policies that aim at preventing or reducing the impact of adverse events will need to target multilevel changes
		Score	+++	+	+++			++	+++	+++
25	McGoldrick: 2007	Comment	Expert essay	USA	NA	To discuss Guideline's recommentations implementation on the management of MDRO in home setting	NA	no info	no info	- Administrative measures (ICP: annual review)<br>- staff education and training<br>- Surveillance system (M&E)<br>- Prevention and control (hand hygiene: SOPs: contact precautions: equipemnt and supplies: environment)
		Score	+	+	-			-	-	++

26	Miliani: 2015	Comment	Point prevalence study	France	all age groups	To describe the major characteristics of HAIs and antibiotic consumption in HBHC and to identify risk factors associated with HBHC- associated infections	different HAI	HBHC-associated infections were those occurring in a patient during the process of care: neither present nor incubating at the time of starting home care (Day 1): for which the signs and symptoms became apparent after Day 2 and were not associated with a previous discharge from an HCF.<br>(HAI defined using ECDC's case defniton)	- Urinary catheter<br>- At least one vascular catheter<br>- A McCabe score 1 or 2	- Start understanding the epidemiology of HAIs in HHC<br>- Programme surveillance initiatives<br>- Train ICP and general HC staff<br>- Raise awarness<br>- Empower patients
		Score	+++	++	+++		+++	+++	+++	+++
27	Morrison: 2004	Comment	Expert essay	Canada	NA	To develop a model upon which the resources required to support an effective: integrated infection prevention and control program across the health care continuum could be based	NA	no info	no info	Critical infection prevention and control in home care:<br>- Coordination of care as patients move from<br>institutional to other health care sectors.<br>- Communication: including a centralized database<br>and information system.<br>- Systematic surveillance of infectious diseases using surveillance standards. (Need to develop data collection and program evaluation tools.)<br>- Community consultation.<br>- Outbreak management.<br>- Infection prevention and control education of formal and informal (i.e.: family members) caregivers: including development of resource material. Orientation and continuing education of staff.<br>- Marketing of basic infection prevention and control measures e.g.: handwashing.
		Score	+	+	-			-	-	++
28	Moreau: 2002	Comment	Observational study	USA	all age groups	To document the complications associated with CVC use in a home care	CVC	Catheter infection was defined as an infectious event involving the intravenous catheter documented through laboratory findings (eg: positive blood and catheter cultures). It can be either a local site infection or a systemic primary BSI.	Identified as more common characteristic but there is no statistical test: so we cannot consider the results as strong evidence	Management of catheter complications through early identification: education: prevention: and timely treatment can result in cost containmentcontainment in hospital and outpatient settings
		Score	++	+	+++		+++	+++		
29	Patte: 2005	Comment	Point prevalence study	France	all age groups	To estimate the prevalence of HAIs in the home care setting: to determine potential risk factors for infection: and to increase staff awareness of HAIs	different HAI	Uses CDC defintions for nosocomial infections (1988)	Multivariable analysis:<br>-Advanced age<br>- HHC >30 days<br>- Low Karnofsky index<br>- Urinary catheter<br>- Skin wound	The authors consider the study as a source of insights that serve as a basis for imprving surveillance and prevention
		Score	+++	++	+++		+++	++	+++	
30	Poff: 2014	Comment	Expert essay	USA	NA	To share how one agency built a meaningful IC program.	different HAI	Mentions that used APIC accepted definitions	no info	- HHC IC program should focus on the patients or services that are of greatest risk for home care<br>- Track infections that are a direct result of care or services provided by home care and/or identify those

										that could be decreased by interventions under the control of home care staff<br>- track those infections that occur 3 days after hospital discharge and thereby not erroneously identifying them as HAIs<br>- HHC is in need of standardized tracking and benchmarking
		Score	+	+			+++	++	-	++
31	Popp: 2006	Comment	Qualitative observational study	Germany	all age groups	To assess the implementation of the IC guidelines in home care	different HAI	no info	Qualified infection control staff is lacking: infection control protocols are lacking or are not adapted: the cooperation with family doctors often is problematic: there are deficits in hand hygiene and great deficits with clothing hygiene and waste disposal.	Suggest improvements for some care tasks: e.g.: handling of urinary catheters: infusions and prescription of tracheostomy tubes.
		Score	++	++	+++		+++	-		
32	Rinke: 2013	Comment	Telephone survey	USA	children	To investigate HHC agency CLABSI definitions and prevention policies and compare them to the Joint Commission National Patient Safety Goal: the CDC CLABSI prevention recommendations: and a best-practice central line care bundle for inpatients	CLABSI	APIC definition	The article does not indetify or report risk factors but mentions that in children they are different than in adults	Standardization of an ambulatory CLABSI definition and continued oversight of home health care agency consistency with national recommendations is needed
		Score	++	+	++		+++	++	+	+++
33	Szeinbach: 2014	Comment	Retrospective chart review	USA	>18 years	To describe catheter complications and outcomes in patients who received HPN therapy	CLABSI	CLABSIs were defined as an infection that was documented in the patient charts as a catheter-related infection based on clinical evidence and quantitative blood culture data when appropriate. Secondary BSI attribute to a secondary source were excluded	- Number of days of HPN (for all complications: including CLABSIs)	- Additional research is needed<br>- A heuristic model for risk assessment including educational workshops should be considered to help caregivers and clinicians identify patients that may be more susceptible to catheter complications
		Score	++	+	++		+++	+++	+++	+++
34	Saqui: 2007	Comment	Prospective cohort study	Canada	all age groups	To determine the rate and types of bloodstream infections in a Canadian HPN program	CVC	From CDC: (1) laboratory-confirmed BSI: bacterial or fungal organisms isolated from blood culture: when no apparent infection was occurring at another site; and one of fever (>38 degrees Celsius): chills: or purulent discharge at CVC site; (2) clinical sepsis due to CVC; fever or chills and no or negative blood culture and no apparent infections were occurring at another site; and (3) catheter-related infection: purulent discharge: or erythema (>18 mm) at CVC site or along CVC tunnel site: and no or negative blood culture.	no info	no info
		Score	+++	+	+++		+++	++	-	-
35	Schantz: 2001	Comment	Expert essay	USA	NA	Reports the experience of a big HHC agency in	CVC: CAUTI	**For CVC**: one of the following**:**	no info	Development of an IC surveillance system to test definitions: establish

						IPC programs and its collaboration with CDC and APIC		1. Two or more of the following signs/symptoms are present:<br>• Erythema associated with the central line<br>• Pain associated with the central line<br>• Purulent drainage from the exit site<br>• Elevated serum white blood cells<br>• Fever of 100.4F or greater<br>• A pathogen or pathogens are cultured from the CVC<br>2. A physician prescribes a course of antibiotic treatment<br>for a suspected or confirmed central venous catheter<br>infection.<br>3. The physician orders the CVC to be pulled due to suspected or confirmed infection.<br>**For Bladder Catheter**: one of the following:<br>1.There is a change in the character of the urine (hematuria: increasing levels of sediment: foul odor) and two or more of the following signs/symptoms are present:<br>• Fever of 100.4 or greaterb<br>• New flank pain or suprapubic painb<br>• Elevated serum WBCb<br>• Worsening or change in mental statusb<br>• A pathogen or pathogens are cultured from the urine<br>2. A physician prescribes a course of antibiotic treatment for a suspected bladder infection (exclude prophylactic or maintenance antibiotics)		infection baselines: and develop tools for home health agencies in order to improve patient outcomes
		Score	+	+				+++	-	+++
36	Shang: 2018	Comment	Expert essay	USA	NA	Reports the state of art on the agenda for infection prevention and control in HHC	different HAI	no info: refers to APIC definitons	no info	- Previous estimates of infections in HHC are likely to underestimare the problem<br>- increasing HHC services will require better understanding of the topic<br>- Guidelines are based on experts opionin and less research<br>- Need for better understandig of AMR in HHC
		Score	+	+			+++	-	-	
37	Sienkiewics: 2008	Comment	Prospective cohort study	USA	>18 years	To describe the development of a UTI benchmarking project by the Home Care Association of New Jersey for New Jersey's home care agencies.	CAUTI	use definition APIC	urinary catheterization	- Use standard definitions<br>- Share information between agencies<br>- Breaking old habits is difficult but possible with evidence to support the change in practice<br>- Research for appropriate use of antibiotics ofr UTIs
		Score	+++	+	++		+++	++	++	+++
38	Spires: 2018	Comment	Retrospective study	USA	>18 years	To understand the burden of mechanical	CLABSI	CLABSI defined utilizing the National Healthcare Safety Network definition	- female patients<br>- patients with a CVC with >1	- emphasize the need for improved formal surveillance mechanisms in

						complications and CLABSIs in the outpatients setting and to elucidate their impact on the healthcare system		of CLABSI as described in January 2014 protocols with a modified timeframe shifting the date of the event to be on or after day 3 of discharge to identify events in the outpatient setting	lumen<br>- patients receiving TPN	outpatients with CVCs<br>- Further study and formal surveillance of patients in the outpatient setting would help elucidate modifiable risk factors for CACs and CLABSIs
		Score	++	+	++		+++	+++	+++	+++
39	Dibb: 2017	Comment	Expert essay	UK	NA	Overview of complication in cetheterized patients in HPN	CRBSI	Mentions definitions from CDC: IDSA and ESPEN	- Catheter related venous thrombosis<br>- having had a CRBSI increases the risk of getting another CRBSI	- The clical diagnosis (with symptoms) can lead to overestimation of infection and overuse of antibioitcs<br>- develop robust catheter care protocols: supported by specialist nurses<br>- training of multidisciplinary teams<br>- proactively search for and treat CRBSI
		Score	+	++			+++	++	++	++
40	Donaghy: 2014	Comment	Survey on home nurses	France	NA	To identify key areas of concern in home care nursing practice with a view to improving patient safety and infection control practices	different HAI	no info	- Compliance with legal requirements for clinical waste management<br>- Availability of personal protective equipment<br>- lack of hospital liaison when the patients are discharged	Act on the areas of main concern for IPC to improve quality and safety of home care nursing:<br>- Access to continued professional development<br>- Cost and quality of single use materials<br>- Management of clinical waste<br>- Limited prescribing rights<br>- Poor communication between hospital and home care nurses
		Score	++	++						
41	Rhinehart: 2001	Comment	Expert essay	USA	NA	Overview of the state of art at the time and advocacy for surveillance and IPC specific to home care	different HAI	Proposes definitions: see full text. Written before the APIC definitions7	- age<br>-chronic illness<br>- immunosuppression	- need for studies to understand the risk factors<br>- to see if the empiric approach is effective<br>- hospital based IPC professionals must support and guide the home care collegues to develop evidence-based approach
		Score	+	+			+++	+++	+	+++
42	Dressen: 2013	Comment	Systematic review	Belgium	>=17 years	To provide an overview of CRI rates: causative pathogens and associated risk factors	CRI	no info: refers to CRI generic guidelines which apply to all settings	**Device related:**<br>- number of lumens<br>- type of vein canulated<br>- infusion ports more than tunneles external catheters<br>- PICCs more than other centrally placed venous access devices<br>- locking with heparine as with normal saline with ethanol<br>- locking with taurolidine is protective<br>**-Education related:**<br>- training of patients<br>**Follow-up factors:**<br>- being close to a HPN centre<br>- higher number of dependants<br>- if family member takes care<br>**Patient related:**<br>- underlying disease<br>**Time related:**<br>- more than 7 days HPN	- Use standard definitions<br>- training of patients<br>- use tunneled external chateters<br>- lock with taurolidine intead of heparin<br>- standardise the definitions<br>- register the infections properly

		Score	+++	+++	++		+++	-	+++	+++
43	Santrapia: 2016	Comment	Retrospective observational study	Italy	>18 years	To evaluate:<br>1) CVC infection rate and the type of infectious agent determining CRBSI in a patients in HPN<br>2) predictive risk factr of CVC infection and effectiveness antibiotic therapy	CVC	ESPEN and IDSA guidelines: CRBSI is defined as isolation of the same microorganism from semi-quantitative or quantitative cultures of both blood drawn from the catheter lumen and the blood peripherally drawn of the patient with clinical symptoms of a bloodstream infection and no other apparent source of infection.	- Previous catheterization<br>- presence of an enterocutaneous stoma	no info
		Score	++	++	++			++	+++	-

AE: Adverse event; **AMR**: Antimicrobial resistance; **APIC**: Association for professionals in infection control and epidemiology; **BSI**: blood stream infection; **CAUTI**: Catheter-associated urinary tract infection; **CDC**: Centers for disease control and prevention; **CRBSI**: Catheter-related bloodstream infection; **CRI**: Catheter-related infection; **CVAD**: Central venous access devices; **CVC**: Central venous catheter; **ED**: Emergency department; **EPA**: United States Environmental Protection Agency; **ESI**: Exit site infection; **ESPEN**: European society from clinical nutrition and metabolism; **HAI**: Healthcare associated infection; **HBHC**: Home based hospital care; **HC**: Health care; **HHC**: Home health care; **HPN**: Home parental nutrition; **HSCT**: Hematopoietic stem cell trans- plantation; **IC**: Infection control; **ICP**: Infection prevention and control; **IDSA**: Infectious Diseases Society of America; **IV**: Intravenous; **M&E**: Monitoring and evaluation; **MDRO**: Multidrug-resistant organisms; **NA**: Not applicable; **OPAT**: Outpatient parenteral antimicrobial therapy; **PD**: Peritoneal Dialysis; **PICC**: Peripherally inserted central venous catheter; **PN**: parenteral; **PPS**: Point prevalence survey; **SOP**: Standard operating procedures; **RAI**: Resident Assessment Instrument; **TPN**: Total parenteral nutrition; **UK**: United Kingdom; **USA**: United states of America; **UTI**: Urinary tract infection; **VAP**: Ventilator-associated pneumonia.

Supplement VI

Participant's characteristics of HAI in HHC in-depth interviews

Profession/ degree	Gender	Year of HHC experience in present position	Position (relevant for this interview)	Language
1.Medical doctor	F	8	Public health manager at policy level (regional)	FR
2. Nurse	F	14	Public health manager at policy level (regional) – involved in infection prevention	NL
3. Nurse	F	10	Researcher in the field of healthcare associated infections at a federal institute	NL
4. Nurse	M	6	Coordinator for an organisation of HHC provided by nurses– involved in HAH – also still conduct patient home visits	FR
5. Nurse	F	8	Responsible for HAH for an organisation that provides HHC conducted by nurses – also still conduct patient home visits	FR
6. Nurse	M	4	Responsible of team of nurses for an organisation that provides HHC conducted by nurses	FR
7. Nurse	F	-	Staff member at an organisation that provides HHC conducted by nurses – responsible for IPC in HHC – regional level	NL
8. Nurse	F	6	Staff member at an organisation that provides HHC conducted by nurses – responsible for IPC in HHC – provincial level	NL
9. Nurse	M	4.5	Staff member at an organisation that provides HHC conducted by nurses – responsible for IPC in HHC – provincial level	NL
10. Microbiologist	M	-	Coordinating OPAT	NL
11. Microbiologist	F	-	Involved in IPC in hospital + Management group for antibiotic therapy	FR
12. Nurse	F	-	Coordinating OPAT	NL
13. Nurse	F		Coordinates a hospital at home project + provides HHC as a nurse	FR
14. Medical doctor	F	8	GP (in an association with other GPs) conducting home visits + previous experience in academics	FR
15. Medical doctor	F	7	GP (in an association with other GPs) conducting home visits	FR
16. Medical doctor	M	12	GP (in an association with other GPs) conducting home visits	NL
17. Physiotherapist	F	2	Self-employed independently working physiotherapist conducting home visits	FR
18. Midwife	F	3	Self-employed independently working midwife conducting routine pre- and post-partum care at home	NL
19. Nurse	F	5	Self-employed independently working HHC nurse conducting home visits	NL
20. Nurse	F	12	Nurse (working for an association of GPs) conducting home visits	NL
21. Lay healthcare worker	M	-	Family member caring for a patient	FR

D: Dutch; **F**: female; **FR**: French; **GP**: general practitioner; **HAH**: hospital at home; **HAI**: healthcare associated infections; **HHC**: home healthcare; **M**: male; **OPAT**: outpatient parenteral antimicrobial therapy
